# Influence of Proximal, Distal, and Vestibular Frames of Reference in Object-Place Paired Associate Learning in the Rat

**DOI:** 10.1371/journal.pone.0163102

**Published:** 2016-09-22

**Authors:** Lilliana M. Sanchez, Shannon M. Thompson, Benjamin J. Clark

**Affiliations:** Department of Psychology, University of New Mexico, Albuquerque, New Mexico; University of Otago, NEW ZEALAND

## Abstract

Object-place paired associate learning has been used to test hypotheses regarding the neurobiological basis of memory in rodents. Much of this work has focused on the role of limbic and hippocampal-parahippocampal regions, as well as the use of spatial information derived from allothetic visual stimuli to determine location in an environment. It has been suggested that idiothetic self-motion (vestibular) signals and internal representations of directional orientation might play an important role in disambiguating between spatial locations when forming object-place associations, but this hypothesis has not been explicitly tested. In the present study, we investigated the relationship between allothetic (i.e., distal and proximal cues) and vestibular stimuli on performance of an object-place paired-associate task. The paired-associate task was composed of learning to discriminate between an identical pair of objects presented in 180° opposite arms of a radial arm maze. Thus, animals were required to select a particular object on the basis of spatial location (i.e., maze arm). After the animals acquired the object-place rule, a series of probe tests determined that rats utilize self-generated vestibular cues to discriminate between the two maze arms. Further, when available, animals showed a strong preference for local proximal cues associated with the maze. Together, the work presented here supports the establishment of an object-place task that requires both idiothetic and allothetic stimulus sources to guide choice behavior, and which can be used to further investigate the dynamic interactions between neural systems involved in pairing sensory information with spatial locations.

## Introduction

Recent theoretical and experimental work has argued that the recollection of previous experiences is composed of at least three fundamental elements: “what,” “when,” and “where” [1–7]. In other words, an event has a particular temporal relationship with other events, and may have conspicuous spatial and non-spatial components, including the location, who was present, and which objects were encountered. Several behavioral and neurobiological investigations have demonstrated that the elements of past experiences, such as distinct representations of what, where, and when, and associations between these elements, can be evaluated in behavioral tasks in rats which pair specific items (what) and places (where) using a bi-conditional association rule [8, 9]. In this procedure, rats are specifically rewarded when selecting object A only when it appears in location 1, but not in location 2. In contrast, object B is rewarded only when it is encountered in location 2, but not in location 1. Thus, animals are required to select a particular object on the basis of where it is encountered in the environment, often referred to as an object-place paired associate.

The acquisition and retrieval of object-place paired associates is thought to require a distributed network of brain regions including hippocampal, parahippocampal, limbic thalamus, and prefrontal cortical regions [10–15], but the dynamic interactions between these regions during learning and retrieval are poorly understood. Resolving this issue requires an understanding of the precise stimulus sources that contribute to the acquisition and expression of paired object-place associations. Nevertheless, this issue is complicated by the fact that animals can determine their place, or where they are in an environment, on the basis of a diverse set of idiothetic (e.g., motor, proprioceptive, vestibular) and allothetic (e.g., vision, tactile, olfaction) stimuli that can operate in parallel or sequentially during behavior [16–19]. For instance, using an object-place task, Lee and colleagues [15, 20, 21] demonstrated that reductions in the salience of distal visual cues (i.e., those that are located along the walls of the testing environment), by either partially or completely removing the cues, or by increasing the angle between the cues, significantly increased the number of object selection errors made by rats.

There is considerable evidence that animals can also use idiothetic cues to guide spatial localization, especially when familiar landmarks are obscured or when entering new environments [22, 23, 24]. Idiothetic cues can be used to self-localize through a process of path integration or dead reckoning—that is, animals can track their own movements to maintain an internal representation of spatial orientation in relation to a known landmark or home environment [16, 25–28]. The vestibular system, in particular, produces a signal reflecting the velocity of head rotation, which can be integrated over time to derive directional orientation, and disruption of vestibular cues has been shown to impair disambiguation of spatial locations in navigation tasks [22, 23, 29–32]. Whether idiothetic vestibular cues, and an internally derived sense of spatial orientation, plays a role in determining place in paired-associate tasks has not been explicitly tested, but the possibility has been suggested in recent work [9, 12, 32]. For instance, Grieves et al [32] demonstrated that animals rapidly acquired an item-place task when the spatial locations were oriented in widely different directions, but were slower to learn when the locations occupied similar directions. Thus, representations of directional orientation, possibly based on idiothetic vestibular cues, may facilitate the disambiguation of spatial locations in paired-place associate learning.

The purpose of the present study was to test this hypothesis, and establish a procedure, in which both idiothetic and allothetic sources can be manipulated and by which the neurobiological basis of these processes can be evaluated in future work. Here, we describe a paired-associate task similar to the work by Lee and colleagues [9, 33, 34], which utilized an 8-arm radial maze with choice platforms at the end of each arm and a pair of identical objects placed above food wells in two of the arms. In contrast to previous work, however, we trained rats to discriminate between an identical pair of objects presented in 180° opposite arms of a radial arm maze. Thus, animals were required to select a particular object on the basis of distinct maze arm locations, which were also oriented in distinct environmental directions. After animals acquired the object-place rule, a series of probe tests were therefore conducted to evaluate the relative influence of allothetic (distal landmarks) and idiothetic (vestibular) frames of reference in discriminating between the distinct maze arm locations/directions. Because previous studies have shown that local substratal or surface cues related to the maze can control orientation and place localization in dry maze tasks [35–38], we also investigated the role of the proximal maze reference frame in the object-place task. Here we report that rats utilize self-generated idiothetic cues, derived from the vestibular system, to discriminate between maze arms, and when available, can also use local proximal cues associated with the maze. Importantly, and in contrast to previous studies, the absence of altered performance after distal landmark manipulation suggests that idiothetic stimuli provide a robust sources of control in tasks where the maze arms are oriented by 180°. The present study therefore constrains general assumptions regarding the stimulus sources used in item-place tasks, but also establishes an experimental procedure that can be used in future work to better understand the sensory and neurobiological basis of object-place memory in rats.

## Materials & Methods

### Subjects

Subjects were 16 male hooded Long-Evans rats (Harlan, Indianapolis, IN) that were approximately 160 days of age at the beginning of the experiments. All animals were pair-housed in plastic cages on a 12 h light:dark cycle with food and water available *ad libitum*. The Institutional Animal Care and Use Committee (IACUC) at the University of New Mexico approved all procedures for the studies reported here. During pre-training and experiments, rats were place on a restricted food diet to a weight of 90% of free *ad libitum* feeding diet and given access to water *ad libitum*.

### Radial-Arm Maze, Objects, and Environment

**Fig 1:**
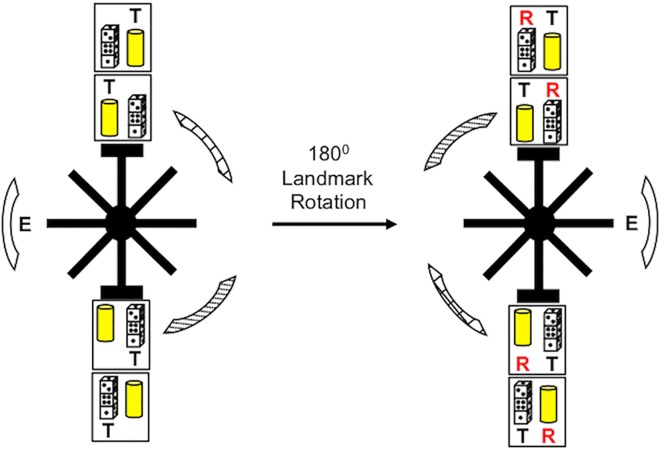
Floor plan with arrangement of room cues, objects, and maze position. Three distal landmarks and experimenter (*E*) position is shown during training (left panel) and probe tests (right panel). *T* indicates the reinforced object-place paired associate during training. *R* indicates the opposite, or reversal, object. If distal cues control choice behavior in the object-place task, then the reversal object will be the preferred choice following 180° rotation of the distal landmarks during the probe test.

A black Plexiglas radial-arm maze was used in all experiments. The maze was composed of eight arms (each 40.1cm × 9.30cm, separated by 45° from each other) that radiated from a center stage (25cm in diameter). The end of each arm was a rectangular platform (20cm × 30cm), each containing three recessed food wells separated by transparent vertical Plexiglas dividers (each 5.1cm × 5.1cm). A transparent Plexiglas door (20cm × 9.5cm) was present at the entrance of each arm from the center stage. A set of toy objects was presented at the end of two opposite arms above recessed food wells. The maze was placed in the center of black circular curtains (5ft in diameter), which was decorated with distinctive visual cues. Two of the visual cues were square foam boards (38 cm x 38 cm); hung approximately 64 cm above the maze, decorated with electrical tape in diagonal and crosshatch designs respectively. The third visual cue was a bed sheet that hung from floor to ceiling along the black curtains, which occupied ~90° of arc. The experimenter stood in front of the floor-to-ceiling bed sheet and also likely served as a visual cue. The configuration of cues was set-up in a triangular formation equally spaced around the curtain as seen in Fig 1. A video camera was positioned above the maze along with a small 100-W incandescent lamp providing illumination.

### Handling and Shaping

All rats were handled for 1–2 weeks for 5 minutes per day. Once rats showed signs of comfort with the experimenter (no defecation/urination), shape training began. Shape training consisted of placing rats in the maze for 20 minutes daily for 7 days with food rewards (quarter pieces of Fruit Loops) scattered across the maze, including two arms of the maze that remained open and the three recessed food wells at the end of each arm. As the rats became comfortable with the maze and consumption of the reward, pieces of food were then strategically removed from the center stage as well as the maze arms and choice platform, however, pieces of food remained in the recessed food wells. Once rats were comfortable retrieving and eating food from the recessed food wells, a metal washer (2.5cm diameter) was placed above the food so they were required to move the washer to retrieve the reward. In this phase of training, rats were given 32 trials per day. A trial consisted of placing a rat in the center stage with all maze arm doors closed, the experimenter then opened one of the doors, the rat ran to the choice platform and displaced the washer to retrieve the reward. The rat was then gently guided back to the center stage to consume the food. While one trial occurred the experimenter quickly re-baited the other arm. After a few days of training, animals began to voluntarily, with little guidance, return to the center stage for consumption, likely due to their natural proclivity to consume large food pieces in enclosed areas of an environment [39, 40]. Once rats were able to displace the washer 32 times within 20min (inter-trial interval of 20–40sec), training in the object-place paired association task began as explained below. The maze was cleaned between animal training sessions. Because the experiment required the rapid removal of maze doors, rebaiting of the maze, and switching of object positions on the choice platform, the experimenter remained in a fixed position within the floor-to-ceiling curtains and next to the maze (Fig 1).

### Object-Place Paired Associate task

Animals were trained in an object-place task [9, 34, 41] in which the washers were replaced with distinct toy objects (a stack of Dice and a yellow tower; see Fig 1). The toy objects were placed above the far left and far right food wells in the choice platform (the middle food well was not used) of two arms located 180° relative to each other. The same two arms were used throughout training. The location of the toy objects in the choice platform were counterbalanced across trials, and rats were required to displace one of the objects to uncover the reward. Thus, animals were required to learn a rule associated with each arm—that is, they had to choose a particular object irrespective of its location in the choice platform (i.e., left or right food well location), but dependent on the location (i.e., the maze arm) of the object. Since accurate object displacement requires an association between a particular place in the maze and object information, this rule is typically referred to as an object–place paired association. An incorrect object choice was punished by preventing the animal from correcting its response and obtaining the food reward. In these cases, the rat was blocked and guided back to the start box with no food reward. The left-right position of objects in the choice platform was pseudo-randomly selected across trials so as to prevent animals from learning a specific egocentric response to obtain the food reward. The sequence of arm visits was randomized with two different sequences that were alternated between days.

### Distal Landmark Rotation

Animals (n = 10) received training in the object-place task as described above for a total of 10 days followed by two probe tests. To discourage the use of proximal cues, the maze was cleaned using a mild solution of soapy water and rotated by 45° between training sessions. The maze was not cleaned between trials, but was cleaned between training sessions for each animal. After 10 days of training, a landmark rotation test was performed in which animals were first tested in the object-place task for 16 trials with the distal cues in the trained configuration (Fig 1). After 16 trials were completed, animals were placed back in their home cages while the investigator rotated the distal environmental landmarks by 180°. Because the experimenter occupied a fixed position next to the maze during acquisition and may have been viewed as a stable extramaze cue [42], the experimenter also rotated their position with the distal landmarks. After approximately 60 min, rats were then returned to the maze where they performed an additional 16 trials of the 180° rotated object-place task. Animals were not presented the food reward on the first trial of the probe test, however subsequent trials were rewarded only if the animal selected the object defined by the distal cues, which for the purposes of clarity in analysis and presentation, we have termed as the *reversal* object (Fig 1). The opposite object, which was reinforced during acquisition, is referred to as the *trained* object. In other words, the *trained* object occupied the same absolute location in the environment across training and probe testing.

### Disorientation

After the landmark rotation test, the same animals were tested in a probe designed to evaluate the use of vestibular cues for maze arm discrimination. Rats first performed the object-place task for 8–16 trials, after which animals were placed in their home cage while the investigator cleaned the maze. In one probe test, the distal cues maintained the same orientation between sessions, but in the second probe test, the distal landmarks were rotated by 180°. For both probe tests, after 60 min, animals were transported individually in an opaque covered plastic box (38cm x 55cm, and 30cm in height) from the colony room to the testing room and were given a disorientation treatment. The disorientation manipulation consisted of the experimenter gently rotating the box in a clockwise and counterclockwise direction for 60sec while walking around the testing room; this was performed before the first set of trials but not between trials. The rate of rotation varied but generally ranged between 90–180° per second. After 60sec, the rats were removed from the box and placed in the center platform, and after several seconds, animals were tested in the object-place task for 8 trials. A similar disorientation procedure has been used in a number of studies [29, 30, 43–48] and was used here to disrupt the accurate tracking of vestibular cues between the holding room and the testing environment.

### Proximal Cue Rotation

Rats (n = 6) were trained in the object-place task for a total of 8 days followed by two probe tests. In contrast to the experiments above, the maze was cleaned, but was not rotated by 45° between training sessions. Thus, in this task, animals could learn to use proximal maze features (shape or visual asymmetries) to disambiguate arm locations. In the first probe test, the landmark rotation experiment was replicated as described above, but the rats were not disoriented. In the second probe test, the landmark rotation experiment was again replicated, but in addition to rotating the distal cues, the maze was also rotate by 180°.

### Data Analysis

An animals object choice was defined as the displacement of an object in the choice platform, which was typically done with the snout, conveniently allowing for an explicit characterization of choice behavior. For each trial, the animals object choice was recorded and the percentage of correct responses was calculated for daily sessions. The percentage of correct object choices during task acquisition was subjected to a repeated-measures analysis of variance (ANOVA) with days as within subject factors. Early in training and prior to learning the object-in-place rule, rats often show a turn-response bias for a particular side of the choice platform [9, 41, 49]. To determine whether the manipulations and testing procedures conducted in the present study encouraged, or discouraged, this temporal expression of a turn-response (left turn vs. right turn), we created a response index bias measure as described in previous studies [9]. The measure was calculated by taking the absolute value after subtracting the number of choices made for the left food well from the number of choices made for the right food well and then dividing the result by the sum of the number of choices. We also calculated the bias index for the correct object-in-place paired associate by subtracting the number of choices made for the incorrect object in the arm from the number of choices made for the correct object, and dividing by the total number of choices [49]. Bias indices for response and object-in-place were subjected to a repeated-measures ANOVA.

On days in which probe experiments were conducted, the preference for the trained object during the 16 baseline trials before and the first 4 test trials after each manipulation was statistically compared using a paired-sample t-test (two-tailed). In addition, Chi-Square tests (*χ*^*2*^) were conducted on probe trials to determine whether object selection was significantly associated with the trained vs. reversal object. Effect sizes for t-tests and ANOVAs were calculated using Cohen’s d (*d*) and partial eta squared (*η*^*2*^), respectively. Statistical tests were conducted using SPSS (23.0, SPSS Inc., Chicago, IL).

## Results

### Do rats use distal landmarks to discriminate maze arm locations in the object-place task?

**Fig 2:**
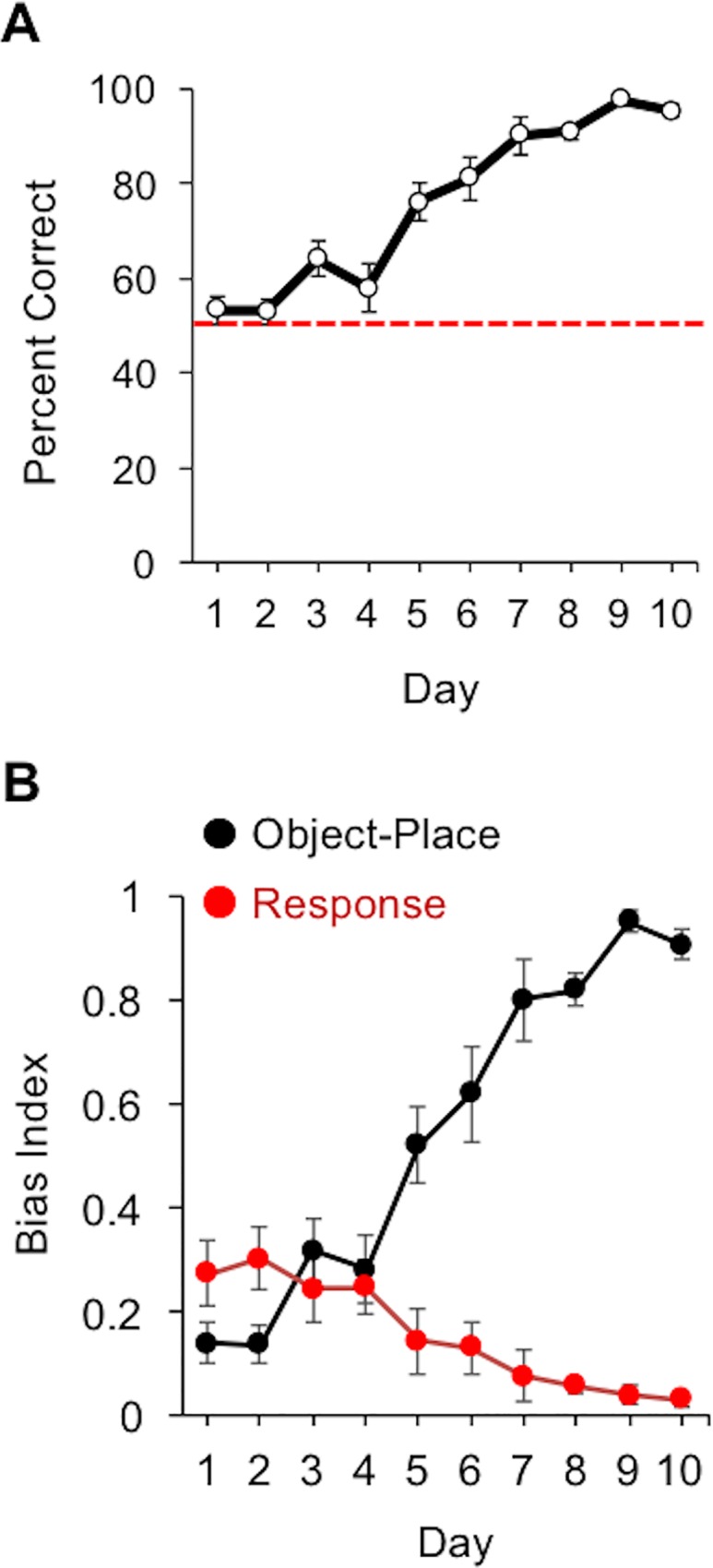
*A*. Plot showing the mean and SEM for the percentage of correct object selections as a function of training day for animals tested in the distal landmark rotation and disorientation probe tests. The dashed horizontal red line indicates chance performance. *B*. Plot showing the mean and SEM for the bias index measured for object-in-place (black) and turn-response (red) behaviors.

Fig 2A plots the percentage of correct choices in the object-place task over the 10 days of training. On average, animals showed marked improvement in selecting the reinforced object, and by Day 10 of training, animals demonstrated a high degree of discrimination between the two objects based on maze arm location (mean ± standard error of the mean: 95.3 ± 1.34%; see also S1 Table). This observation was confirmed by an significant ANOVA yielding a training day effect for percent correct, *F*(9, 81) = 43.8, *p* < 0.001, *η*^*2*^ = 0.83. We also observed an inverse relationship for bias measures of object-in-place and response strategies (Fig 2B). Consistent with previous studies [41, 49], the bias toward performance of a turn-response observed early in training was largely diminished by the end of testing (Day 1: object-place bias: 0.14 ± 0.04; turn-response bias: 0.27 ± 0.06; Day 2: object-place bias: 0.14 ± 0.04; turn-response bias: 0.30 ± 0.06), but the preference for the target object associated with the maze arm significantly increased with training. A repeated-measure ANOVA confirmed this observation with a significant day-by-strategy effect, *F*(9, 144) = 37.8, *p* < 0.001, *η*^*2*^ = 0.70.

**Fig 3:**
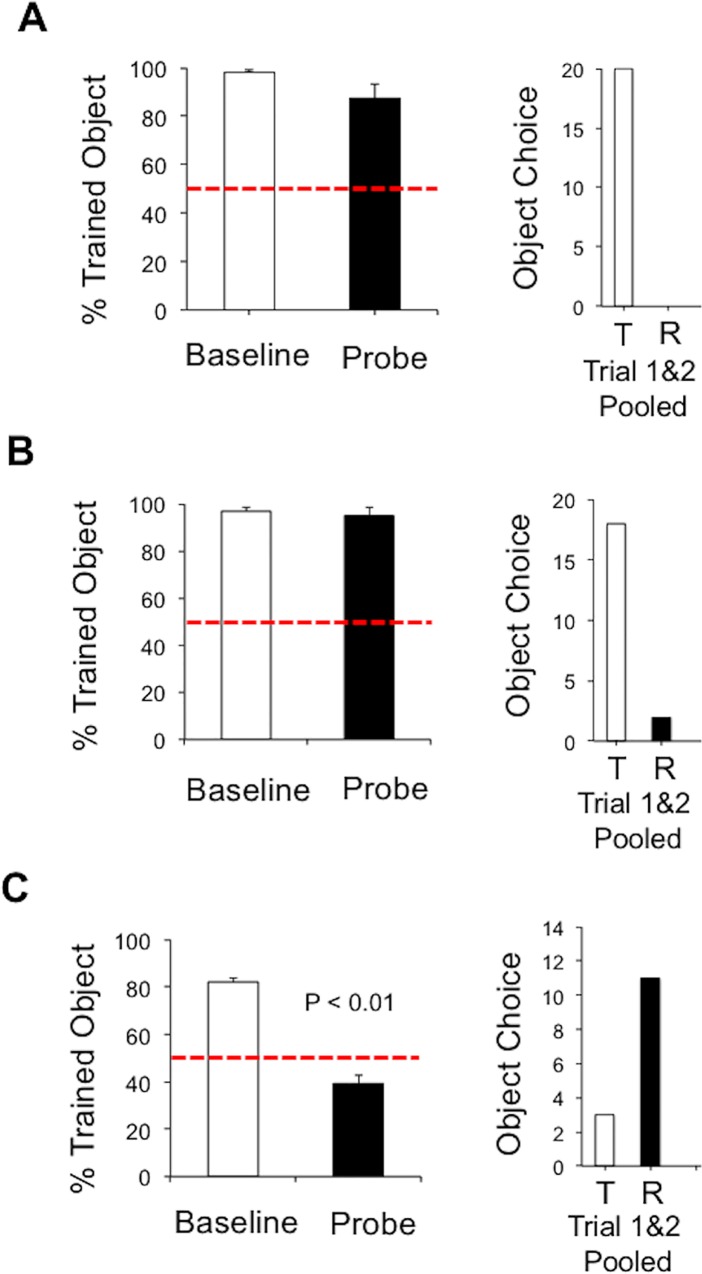
Left panel plots show the mean ± SEM for percent correct (i.e., selection of the trained object) before and after, *A*, distal landmark rotation, *B*, disorientation, and *C* disorientation/landmark rotation. The dashed horizontal red line indicates chance performance. Note that only disorientation/distal landmark rotation resulted in a significant difference between the baseline and the probe trials. Right panels plot the pooled object choice (T, trained object; R, reversal object) across the first two trials of the probe session. Notice that in standard landmark rotation (A) the trained object is preferred, but when the maze is rotated (B), the reversal object (R) is preferred.

We next asked whether animals utilized distal cues to disambiguate maze arm locations in the paired associate task. Thus, on Day 11, animals were given 16 training trials in the object-place task with the distal cues in the standard location, followed by 16 trials with the distal cues rotated by 180°. We hypothesized that if the rule guiding the paired object-place associate is exclusively determined by distal cues, then animals should reverse their object preference following distal cue rotation (see Fig 1). Fig 3A (left panel) plots the percentage of responses made toward the trained object before and after distal cue rotation. On average, in the first four trials after the rotation of the distal cues, animals showed a strong preference for the trained object, which was comparable to the 16 trial baseline training session (Before Rotation: 98.1 ± 1.33%; After Rotation: 87.5 ± 5.59%; *t*(9) = 1.90, *n*.*s*.), suggesting that the change in spatial location of the distal cues had little effect on the object-place behavior of the rats. This was particularly apparent in the first two trials after distal cue rotation as animals displayed an unambiguous preference (i.e., 20 out of 20 trials) for selecting the trained object rather than the reversal object (Fig 3A, right panel). Interestingly, preference for the trained object was generally maintained in trials 3 and 4 (15 out of 20 trials; *χ*^*2*^(1) = 5.00, *p* < 0.05), despite the fact that only the reversal object was reinforced during the probe session. Together, these observations strongly support the conclusion that rats do not preferentially use distal landmarks to define locations within the maze.

### Do rats use vestibular cues to discriminate maze arm locations in the object-place task?

The results above suggest that animals do not exclusively use distal landmarks in this task, suggests that perhaps animals do not make use of distal landmarks in this task, or, alternatively, that other stimuli additionally contribute to the disambiguation of maze arm locations and come to control behavior when the distal cues are manipulated. Because the maze was cleaned and rotated between sessions, proximal cues were unlikely to provide disambiguating information. Thus, we hypothesized that animals may have utilized idiothetic cues such as vestibular information as a disambiguating stimulus. We tested this possibility on Day 12 and Day 13 by first training animals in the object-place task with the distal cues in the standard trained orientation (see Fig 1 for experimental procedures). Following baseline training, animals were disoriented before being placed back in the maze. On Day 12, the distal cues remained in the same orientation between baseline training and the probe, but on Day 13, the cues were rotated by 180°. We reasoned that disoriented rats would be more likely to utilize distal cues for orientation and maze arm disambiguation, and therefore would reverse their object preference in the probe test on Day 13.

Fig 3B plots the percentage of responses made toward the trained object before and after disorientation on Day 12. Similar to the results of the landmark rotation test, animals showed a strong preference for the trained object after disorientation, which was comparable to baseline testing (Before Rotation: 96.9 ± 1.40%; After Rotation: 95.0 ± 3.33%; *t*(9) = 0.52, *n*.*s*.). Again the preference for the trained object largely dominated the first two trials of the probe test (*χ*^*2*^(1) = 12.80, *p* < 0.001; Fig 3B, right panel). On Day 13 baseline testing, 7 of the 10 animals clearly displayed a preference for the trained object (82.1 ± 2.52%), while 3 rats failed to exceed 50% accuracy and were therefore at chance performance. It is possible that these 3 rats were impacted by the previous sessions extinction trials, and because subsequent probe performance would produce difficulties in interpretation, these animals were excluded from further analyses and probe testing. Fig 3C plots the percentage of responses made toward the trained object before and after combined distal cue rotation and disorientation. In contrast to the results of the previous experiments, rats generally reversed their object preference in the first 4 trials of the probe session (After Rotation: 39.3 ± 9.22%; *t*(6) = 3.83, *p* < 0.01, *d* = 0.84; see also S2 Table). This preference was expressed most prominently in the first two probe trials as 11 out of 14 trials were directed toward the reversal object (*χ*^*2*^(1) = 4.57, *p* < 0.05; Fig 3C, right panel). In contrast, preference for the reversal object was reduced to approximately chance levels in trials 3 and 4 of the probe session with only 6 of 14 trials being directed toward the reversal object (*χ*^*2*^(1) = 0.29, *n*.*s*.). This latter observation is particularly striking provided that rats were reinforced for selecting the reversal object, and indicates that disorientation only had a transient influence over spatial localization in this task.

### Do rats use proximal cues to discriminate locations in the object-place task?

**Fig 4:**
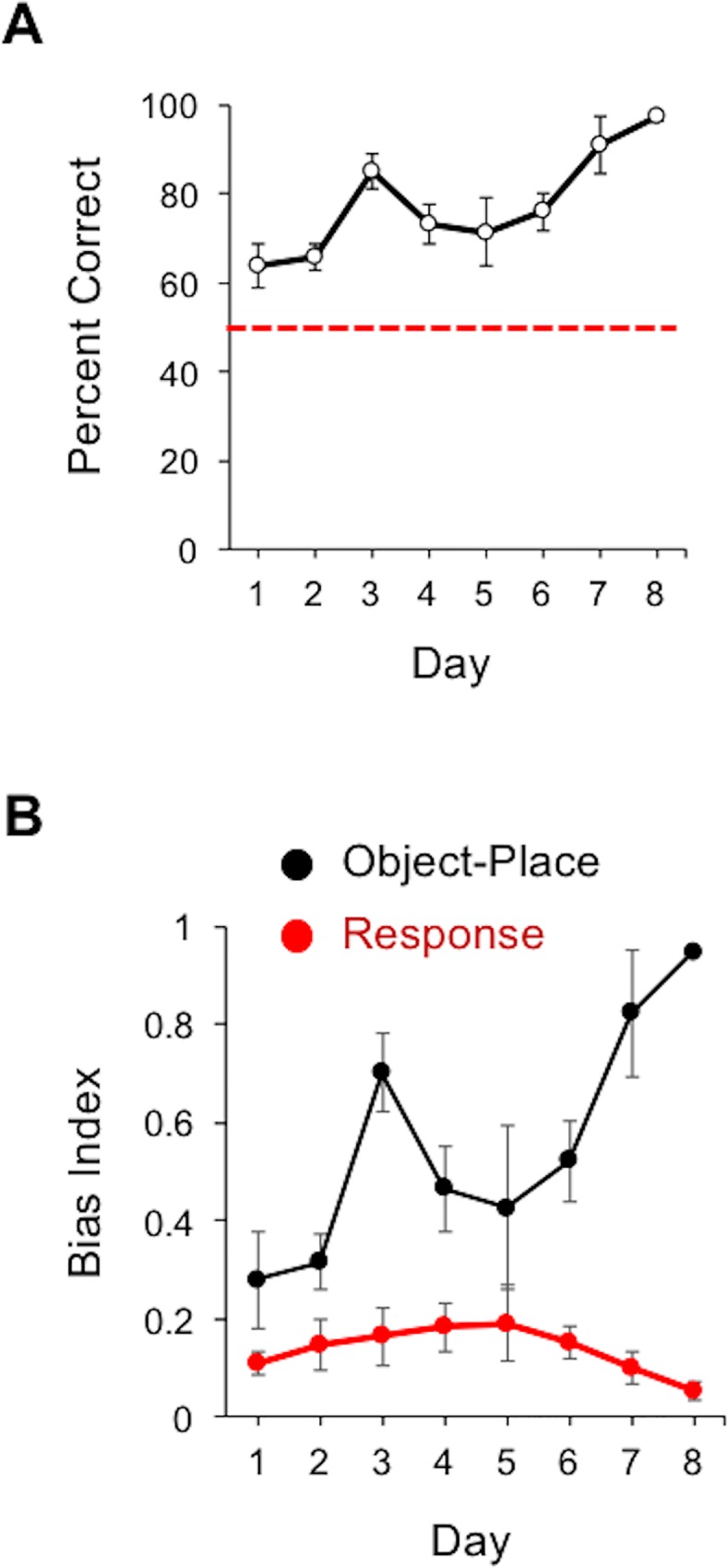
*A*. Plot showing the percent correct (mean ± SEM) across training for animals tested in the distal landmark and proximal cue rotation probe test. *B*. Plot showing the mean and SEM for the bias index measured for object-in-place (black) and turn-response (red) behaviors.

The results of the experiment above suggest that when vestibular cues are disrupted, animals can utilize distal landmarks to disambiguate maze arm locations. This observation suggests that animals can rapidly switch between stimulus sources when one cue is no longer informative or is in conflict with other sensory systems. Nonetheless, the preference for the reversal object was maintained only in the first two trials of the probe test indicating that non-vestibular, non-distal, based cues may have provided conflicting information and may come to control behavior when they are salient. This led us to test the possibility that proximal cues related to the maze substrate could potentially be used to disambiguate arm locations in this task. A group of 6 naïve rats were trained over 8 days in the object-place task with the distal cues in the standard orientation. In contrast to the experiments above, the maze was not rotated between sessions, allowing animals to learn a consistently presented set of local maze asymmetries. Fig 4A plots the percentage of choices in the object-place task over the 8 days of training. An ANOVA yielded a significant training day effect for percent correct, *F*(7, 28) = 7.68, *p* < 0.001, *η*^*2*^ = 0.66, suggesting again, that by the final testing day, animals learned to discriminate between the two objects on the basis of maze arm location (Day 8: 97.4 ± 0.96%; see also S3 Table). We again observed an inverse relationship for bias measures of object-in-place and response strategies (Fig 4B) with a significant interaction between the bias index for turn response and the object-place response (*F*(7, 56) = 7.89, *p* < 0.001, *η*^*2*^ = 0.50). It is noteworthy that with the maze maintaining the same position between sessions, there was no significant bias for turn-response behavior early in training (Day 1: object-place bias: 0.28 ± 0.10; turn-response bias: 0.11 ± 0.02; Day 2: object-place bias: 0.32 ± 0.06; turn-response bias: 0.15 ± 0.05).

**Fig 5:**
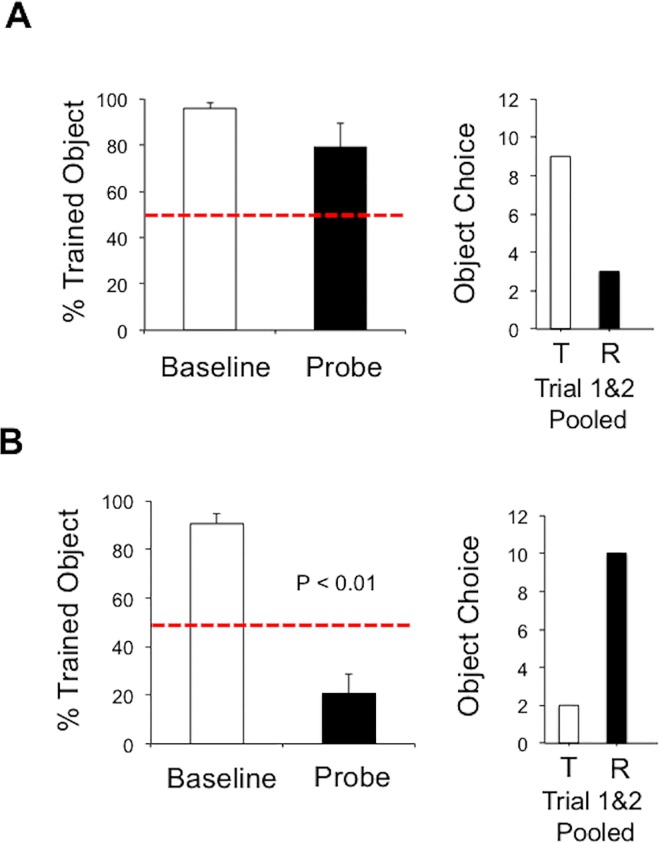
Left panel plots show the mean ± SEM for percent correct (i.e., selection of the trained object) before and after, *A*, distal landmark rotation, *B* maze/landmark rotation. Note that maze/distal landmark rotation resulted in a significant difference between the baseline and the probe trials. Right panels plot the pooled object choice (T, trained object; R, reversal object) across the first two trials of the probe session. Notice that in standard landmark rotation (A) the trained object is preferred, but when the maze is rotated (B), the reversal object (R) is preferred.

On Day 9, we replicated the distal landmark rotation experiment, and animals showed a strong preference for the trained object during baseline testing in the standard distal cue configuration (95.8 ± 2.64%; see Fig 5A). Consistent with our results above, there was a general absence of control by the distal cues as the animals preference was maintained for the trained object during the first 4 trials of the probe test (After Rotation: 79.17 ± 10.0%; *t*(5) = 1.87, *n*.*s*.). On Day 10, we performed the distal landmark rotation, but in addition to rotating the distal landmarks by 180°, we also rotated the maze by 180°. Animals again showed a preference for the trained object during baseline training with the distal and proximal cues in the standard training configuration (90.6 ± 4.18%; see Fig 5B and S4 Table). In contrast to distal landmark rotation alone, rats generally preferred the reversal object during the first 4 trials of the probe session as indicated by a significant reduction in the percentage of responding to the trained object (After Rotation: 20.8 ± 7.68%; *t*(5) = 6.56, *p* < 0.01, *d* = 0.95). The reversal of object selection was particularly prominent in the first two trials (10 out of t12 trials directed toward the reversal object; *χ*^*2*^(1) = 5.33, *p* < 0.05; Fig 5B, right panel), and the preference for the reversal object was generally maintained in trial 3 and 4 of the probe test (10 out of 12 directed toward the reversal object). These observations suggest that the proximal reference frame, in combination with distal cues, provides a strong source of spatial information in the object-place task.

## Discussion

Here we describe a procedure that allows the investigation of the idiothetic and allothetic sensory basis of spatial localization in an object-place task. We suggest that this task can be used to precisely evaluate the neurobiological mechanisms underlying associations between sensory information and spatial stimuli. The present study supports three novel conclusions regarding the influence of idiothetic and allothetic cues to spatial localization in an object-place paired-associate task. First, our findings support the general conclusion that the procedure used in the present study involves the use of vestibular cues in the establishment of object-place paired associations. Specifically, we report that distal landmark rotation produced a reversal in object preference only after animals were disoriented between the animal holding and experimental rooms (see Fig 3). Based on this finding, we argue that disorientation, or rotational stimulation, discouraged the accurate tracking of angular head movements, which would normally allow rats to angular path integrate and maintain an accurate sense of spatial orientation when being placed in the maze [16, 25, 27]. Our conclusion is consistent with other maze studies demonstrating a vestibular basis for self-localization in dry-land and water maze procedures [22, 29, 30, 31], however, we are unaware of work demonstrating the use of vestibular cues in an object-place paired associate task. This work therefore supports previous suggestions that idiothetic cues may provide a fundamental stimulus source in the generation of declarative and episodic memory processing [3, 50], and suggests that a broad network of subcortical and cortical circuits involved in processing vestibular information also contributes to object-in-place memory.

A second conclusion of the present study is that proximal cues associated with the maze substrate can also be used to disambiguate arm locations. This was evidenced by the fact that the coherent 180° rotation of the maze and distal landmarks produced a reversal in object preference, whereas rotation of the distal landmark alone failed to have a similar effect on object selection (see Fig 5). This finding is consistent with work showing that animals can use cues directly associated with the maze substrate for spatial orientation [38], which can include odors [51, 52, 53], or geometric asymmetries in arena shape [35, 54, 55]. Whether rats utilized geometric relationships within the maze, local odor cues, or whether the object nature of the task enhanced the salience of the proximal frame of reference is unclear, and should be investigated in future studies. It is important to note however that the maze was intentionally constructed to avoid asymmetrical features (i.e., 8 evenly spaced arms with identical components on each arm), and we intentionally cleaned the maze surface between rats and testing days with the intention of preventing animals from utilizing olfactory cues. Learning to use specific odors or trails of odors for maze arm discrimination would therefore be difficult between daily sessions, but perhaps possible within a testing session as is suggested by previous work [51, 52, 53]. Also, because only the correct object was rewarded during training, it could be argued that animals may have learned to discriminate between objects based on olfactory cues directly associated with the food reward or directly with the objects themselves. We tested for this possibility in the probe test, in which animals were not rewarded on the first trial, but were rewarded on subsequent trials. Because performance was consistent between the first two trials of each probe session, we suggest that odors directly associated with the reward were not likely used in this task. Further, we have performed tests in which the reward was provided only for a randomly selected subset of trials and found no differences in performance between trials in which food was provided and trials in which it was not provided (data not shown). An additional consideration is that lengthier training schedules are believed to discourage the use of cues associated with places, including distal cues, but encourage response strategies based on motor responses or proximal cues associated with the goal [56, 57]. However, the pattern of results presented in this study, and in previous studies using a similar object-place task [41, 49], suggest that the turn-response biases are preferred early in training rather than later in training (see Fig 2B and Fig 4B).

A final conclusion relates to some key differences between the present work and previous studies investigating the influence of visual contextual cues in object-place learning. In a series of experiments by Lee and colleagues [20, 21], the authors report that discrete changes in visual context, either by completely removing the distal context by turning the room lights off, or by removing a subset of the distal cues, can produce a significant reduction in the percentage of correct object-place choices. Our results however indicate the exclusive or dominate use of distal cues in place discrimination in the object-place task, as landmark rotations failed to produce reversals in object selection. However, our results support the interpretation that the relationship between the distal landmarks and the maze arms were learned during task acquisition, but were utilized only after vestibular and proximal stimulus sources were disrupted. Thus, animals were capable of rapidly switching between stimulus sources to maintain accurate performance. Procedural differences in the present study may have discouraged the exclusive use of distal landmarks, while encouraging the use of multiple stimulus sources for accurate performance. First, it is possible that the distal cues in the present study were not as salient as in previous work. For instance, three distinct landmarks hung along the curtains, and the experimenter was located in a constant position in relation to the distal cues during training and in probe tests (see Fig 1). In contrast to the object-place tasks by Lee and colleagues, and work by others using a radial-arm maze procedure [58], the distal cues in the present study were not located directly behind the choice platform of each arm, and were possibly not clearly visible while animals were on the choice platform. A second consideration is that Lee and colleagues trained rats to perform their object choices on two maze arms that were clearly spatially distinct, but were pointing in the same relative direction within the room reference frame [9]. Thus, it is possible that the ambiguity in maze arm direction may have discouraged the use of vestibular and directional stimulus sources to disambiguate maze arms. This latter possibility is supported by recent behavioral work showing that the acquisition of a paired odor-place associate conducted in adjacent maze compartments is impaired relative to the same paired associate acquired in radially arranged, and opposing, maze compartments [32]. Future work could be directed at systematically manipulating the saliency of each of these stimulus sources and determining the impact on probe test behavior as described in the present study. Regardless, the present work demonstrates that distal cues can be used if vestibular cues are disrupted, supporting the general conclusions of Lee and colleagues that distal contextual cues take part in guiding choice decisions in object-place associates [13, 14].

The dynamic interactions between distal, proximal, and vestibular cues in the performance of object-in-place memory has broad implications with respect to understanding the neural circuitry involved in memory episodic-like memory in rodents. Namely, our findings indicate that neural systems involved in conveying vestibular and directional orientation information also play a role in object-in-place information processing. An attractive hypothesis [9, 32] is that vestibular and directional cues may be provided by head direction (HD) cells, which are neurons found throughout the limbic system that fire as a function of an animal’s heading in an environment [59]. Although HD cells are modulated by a wide range of idiothetic cues such as proprioceptive motor and optic flow cues [60], vestibular information has been shown to be necessary for the expression of HD cell signals as direct lesions of vestibular system circuitry, as well disorientation manipulations, can impair the HD cell signal [48, 61–64]. HD cells are found within several nuclei that are collectively identified as part of the classic Papez circuit, including the anterior thalamus, lateral mammillary nuclei, retrosplenial cortex, and presubiculum [59, 64, 65]. Interestingly, lesions of the anterior thalamus and disruption of neural plasticity in the presubiculum can impair choice behavior in variants of the object-place task [10, 11, 66]. Thus, it is possible that when the direction of reinforcement provides a salient source of discriminative information in object-place learning, HD cell circuitry, through its diffuse limbic projections to the hippocampal formation may influence the formation of paired-place associations such that objects can also be linked with “directions” based on vestibular cues. Previous work has demonstrated that neural pathways involving the hippocampus and other limbic structures have been linked to the acquisition of object-place paired associates [13, 14, 15]. Some recent electrophysiology work supports the possibility of a dynamic interaction between hippocampal place cell and idiothetic processing in a paired associate task [15, 32], in particular, a study by Park and Lee [15] showed that hippocampal place cells generally maintained their location-specific firing despite novel changes in distal cue configurations (i.e., a change in the degree of separation between distal cues), suggesting dominance of the idiothetic frame of reference in guiding place cell orientation. Further, Grieves et al [32] showed that hippocampal place cells were more likely to express non-repeating place fields when the maze arms were arranged in a radial and opposing fashion, but expressed repeating place fields in a maze in which the arms were arranged in parallel. This latter finding may indicate that idiothetic vestibular cues and HD cells provide a discriminating stimulus source for the expression of hippocampal place cell activity.

In summary, the findings from the present study demonstrate three important features of contextual processing in the object-place task. First, the results indicate that animals can determine spatial location in an object-place task based on vestibular information. Second, the results show that animals can utilize local or proximal cue sources related to the maze substrate to localize their place in the environment. Finally, we confirm that distal landmarks are utilized, but are not exclusively used and dominate performance using the procedures in the present study. Instead, we conclude that animals likely use multiple stimuli and can rapidly switch between cues in maintaining accurate performance in object-in-place tasks. Collectively, the results strongly support the general conclusion that a distributed network of subcortical and cortical limbic systems are involved in object-place paired associations, and the object-place procedure used in the present study lends itself uniquely to the determination of the precise sensory mechanisms by which these neural systems may contribute to paired-associate learning.

## Supporting Information

S1 Table The mean and standard error for measures reported in Fig 2. (DOCX)

S2 Table The mean and standard error for the percentage of target object selection reported in Fig 3. (DOCX)

S3 Table The mean and standard error for measures reported in Fig 4. (DOCX)

S4 Table The mean and standard error for the percentage of target object selection reported in Fig 5. (DOCX)
