# Supplementary material for: Influence of Proximal, Distal, and Vestibular Frames of Reference in Object-Place Paired Associate Learning in the Rat
Source: PLoS One. 2016 Sep 22;11(9):e0163102. doi: 10.1371/journal.pone.0163102 (PMC5033391; doi:10.1371/journal.pone.0163102)
Supplement: S1 Table — (DOCX) [file pone.0163102.s001.docx]

|  | Day 1 | Day 2 | Day 3 | Day 4 | Day 5 | Day 6 | Day 7 | Day 8 | Day 9 | Day 10 |
| --- | --- | --- | --- | --- | --- | --- | --- | --- | --- | --- |
| Percent Correct | 53.3  (2.86) | 53.1 (2.72) | 64.1 (3.85) | 57.8 (5.09) | 76.0 (3.99) | 80.9 (4.67) | 90.0 (3.97) | 90.9 (1.58) | 97.5 (1.02) | 95.3 (1.34) |
| Object-Place Bias Index | 0.14  (0.04) | 0.14 (0.04) | 0.32 (0.06) | 0.28 (0.06) | 0.52 (0.07) | 0.62 (0.09) | 0.80 (0.08) | 0.82 (0.03) | 0.95 (0.02) | 0.91 (0.03) |
| Response Bias Index | 0.27  (0.06) | 0.30 (0.06) | 0.25 (0.07) | 0.25 (0.05) | 0.15 (0.06) | 0.13 (0.05) | 0.08 (0.05) | 0.06 (0.01) | 0.04 (0.02) | 0.03 (0.01) |

S1 Table. The mean and standard error for measures reported in Fig 2.
