# Supplementary material for: Influence of Proximal, Distal, and Vestibular Frames of Reference in Object-Place Paired Associate Learning in the Rat
Source: PLoS One. 2016 Sep 22;11(9):e0163102. doi: 10.1371/journal.pone.0163102 (PMC5033391; doi:10.1371/journal.pone.0163102)
Supplement: S2 Table — (DOCX) [file pone.0163102.s002.docx]

|  | Baseline | Probe |
| --- | --- | --- |
| Distal Landmark Rotation | 98.1  (1.33) | 87.5 (5.59) |
| Disorientation | 96.9  (1.40) | 95.0 (3.33) |
| Disorientation and Distal Landmark Rotation | 82.1  (2.52) | 39.3 (9.22) |

S2 Table. The mean and standard error for the percentage of target object selection reported in Fig 3.
