# Supplementary material for: Influence of Proximal, Distal, and Vestibular Frames of Reference in Object-Place Paired Associate Learning in the Rat
Source: PLoS One. 2016 Sep 22;11(9):e0163102. doi: 10.1371/journal.pone.0163102 (PMC5033391; doi:10.1371/journal.pone.0163102)
Supplement: S3 Table — (DOCX) [file pone.0163102.s003.docx]

|  | Day 1 | Day 2 | Day 3 | Day 4 | Day 5 | Day 6 | Day 7 | Day 8 |
| --- | --- | --- | --- | --- | --- | --- | --- | --- |
| Percent Correct | 64.0  (5.02) | 65.8 (2.80) | 85.1 (4.07) | 73.3 (4.39) | 71.4 (7.70) | 76.1 (4.12) | 91.1 (6.43) | 97.4 (0.96) |
| Object-Place Bias Index | 0.28  (0.10) | 0.32 (0.06) | 0.70 (0.08) | 0.47 (0.09) | 0.43 (0.17) | 0.52 (0.08) | 0.82 (0.13) | 0.95 (0.02) |
| Response Bias Index | 0.11  (0.02) | 0.15 (0.05) | 0.17 (0.06) | 0.18 (0.05) | 0.19 (0.08) | 0.15 (0.03) | 0.10 (0.03) | 0.05 (0.02) |

S3 Table. The mean and standard error for measures reported in Fig 4.
