# Supplementary material for: Influence of Proximal, Distal, and Vestibular Frames of Reference in Object-Place Paired Associate Learning in the Rat
Source: PLoS One. 2016 Sep 22;11(9):e0163102. doi: 10.1371/journal.pone.0163102 (PMC5033391; doi:10.1371/journal.pone.0163102)
Supplement: S4 Table — (DOCX) [file pone.0163102.s004.docx]

|  | Baseline | Probe |
| --- | --- | --- |
| Distal Landmark Rotation | 95.8  (2.64) | 79.2 (10.03) |
| Proximal Maze and Distal Landmark Rotation | 90.6  (4.18) | 20.8 (7.68) |

S4 Table. The mean and standard error for the percentage of target object selection reported in Fig 5.
